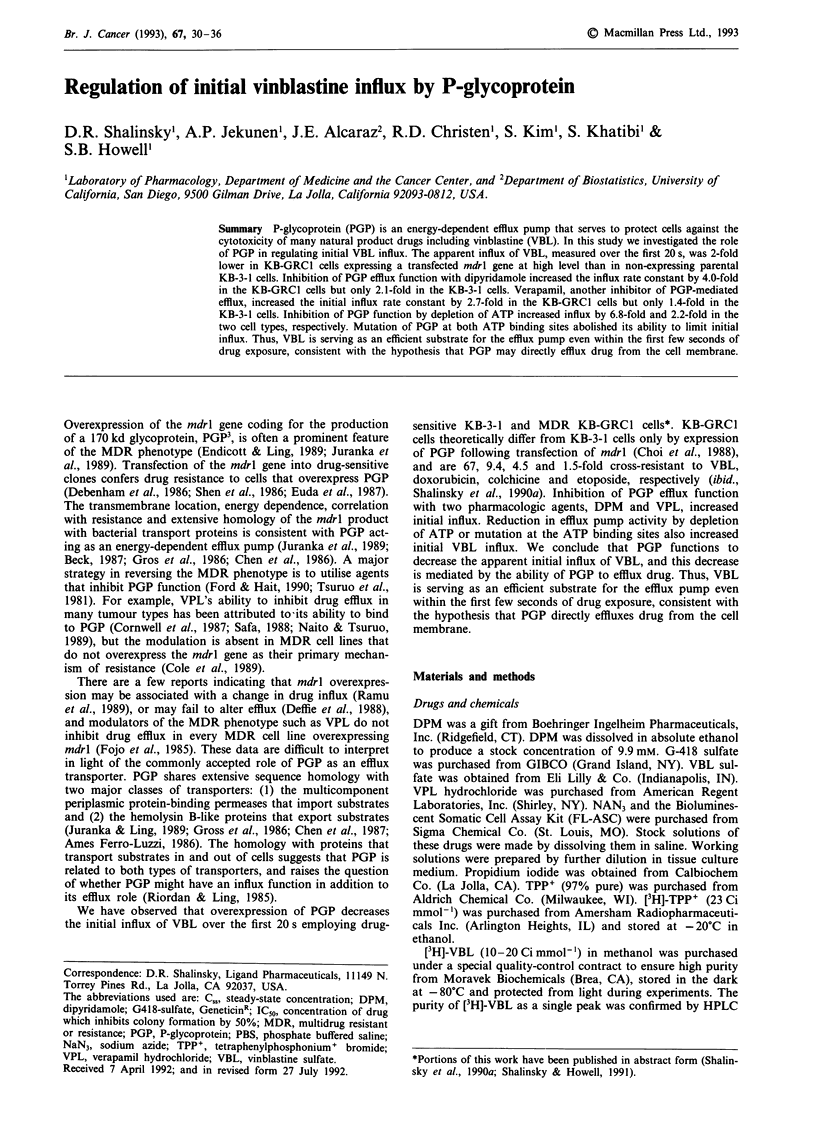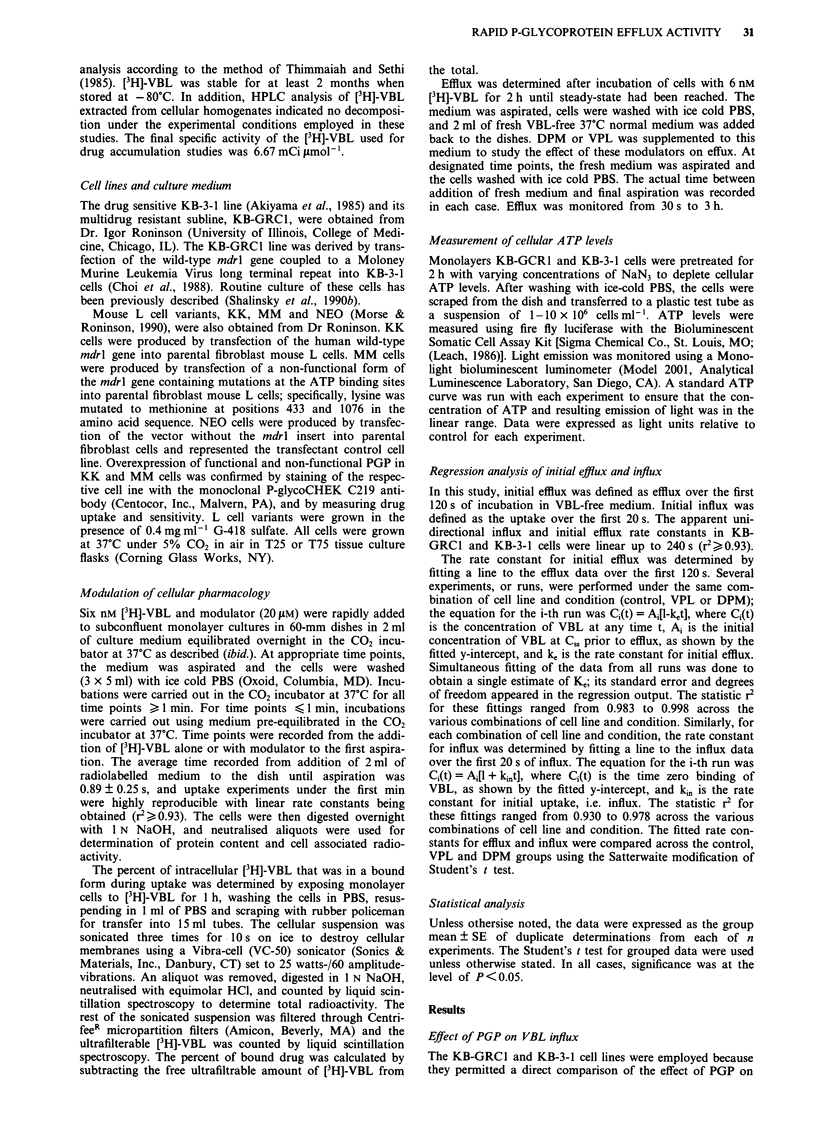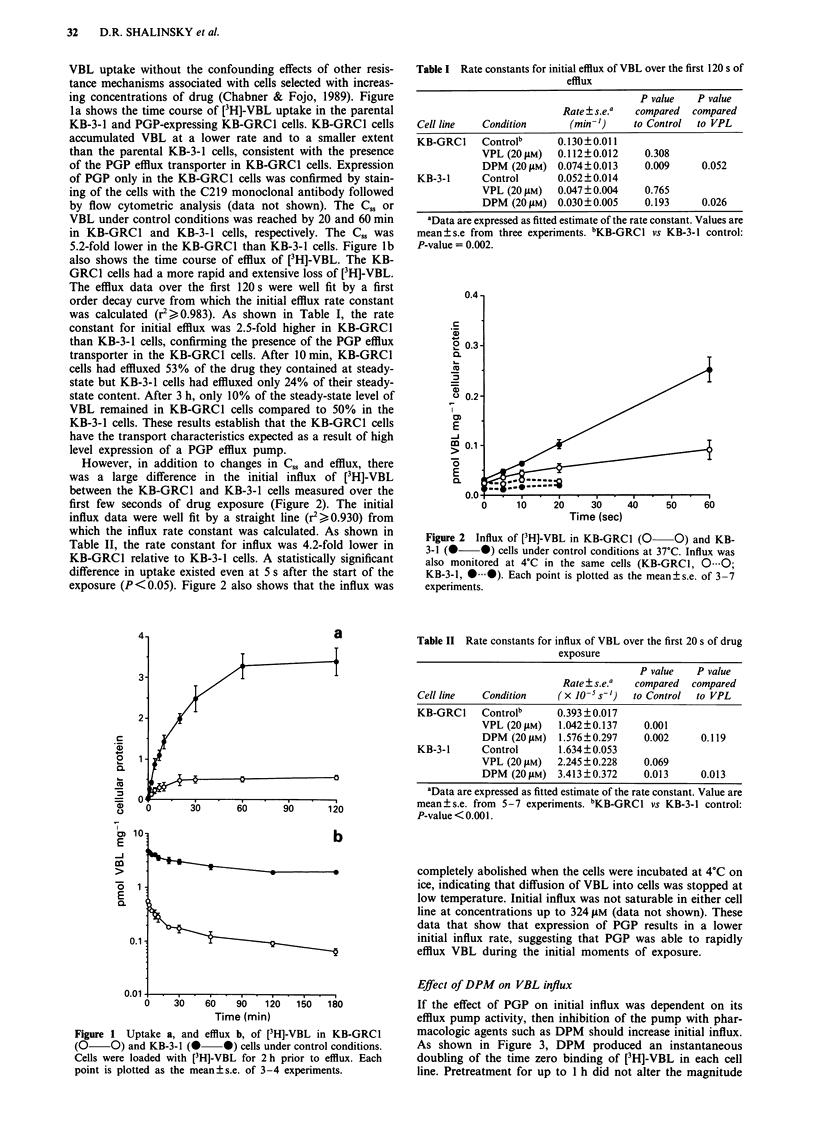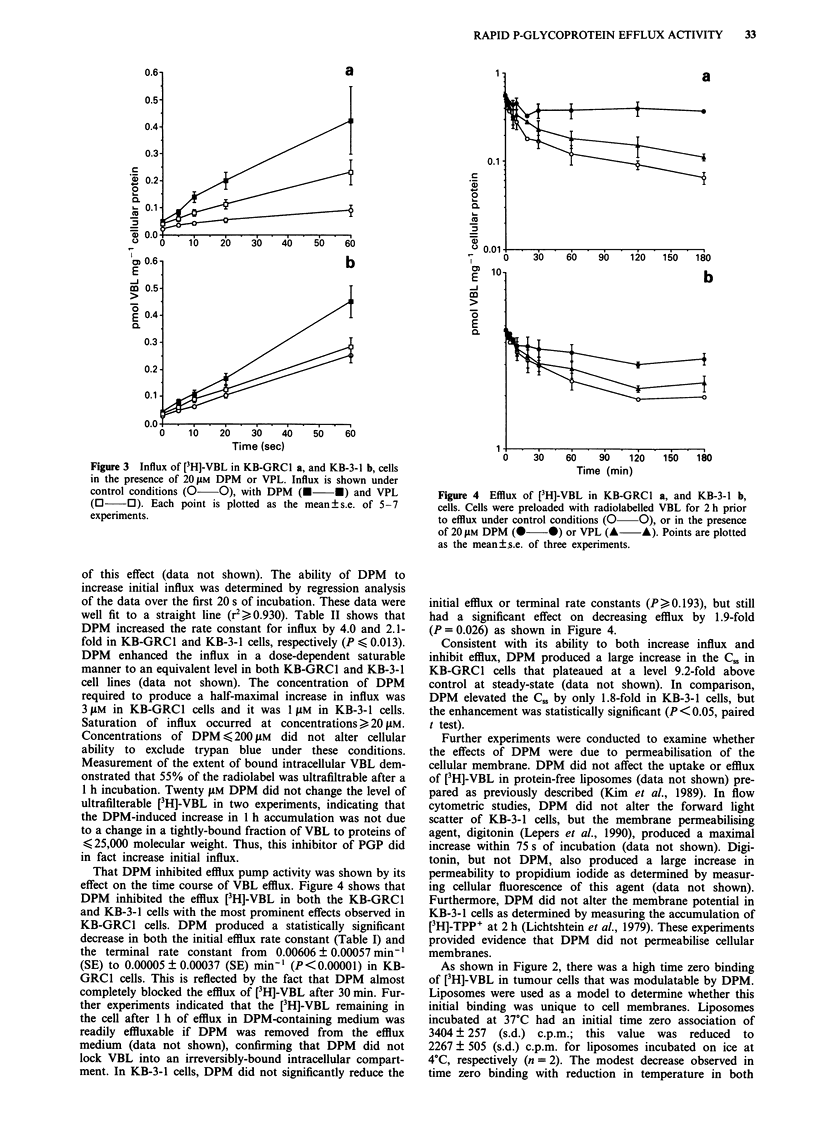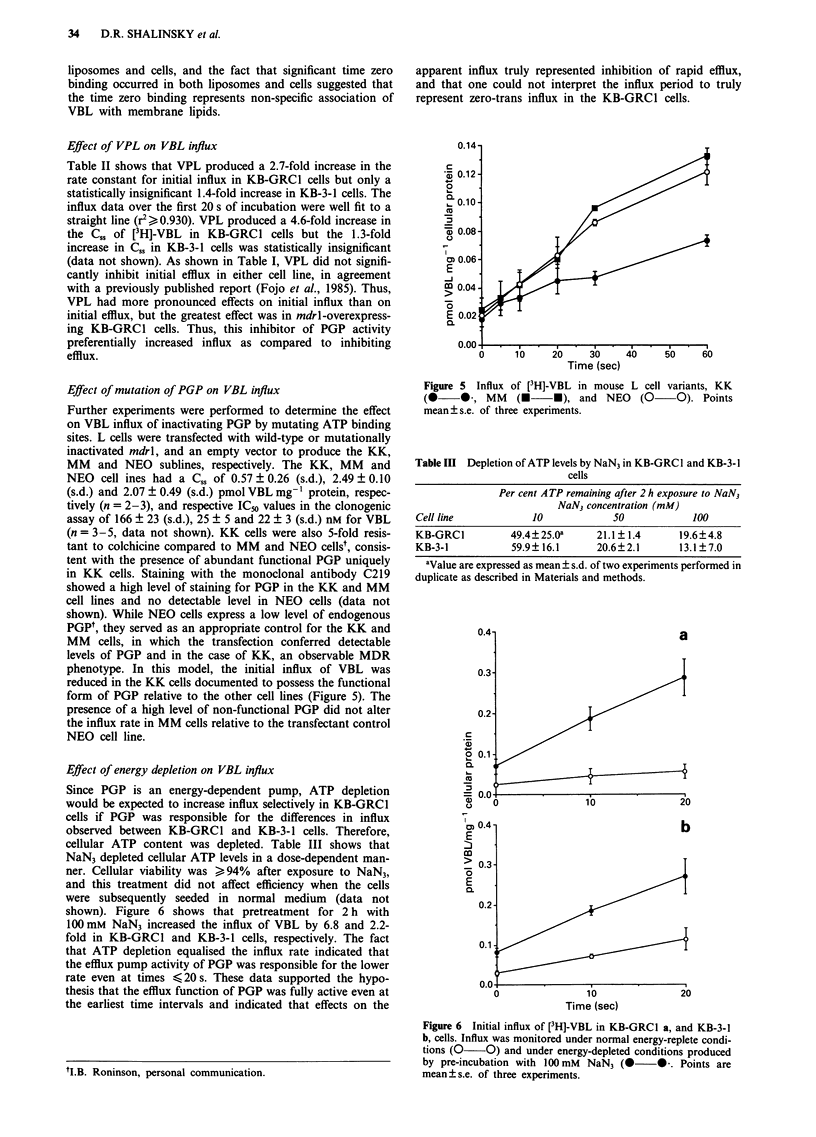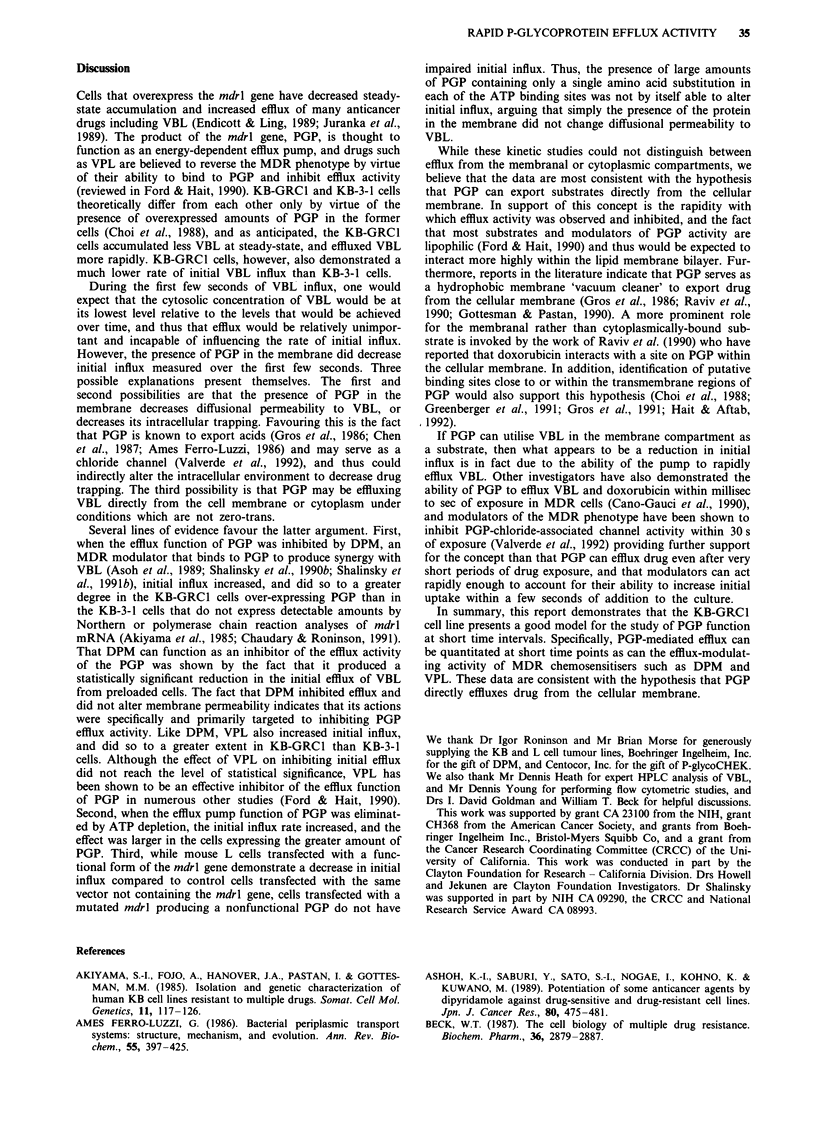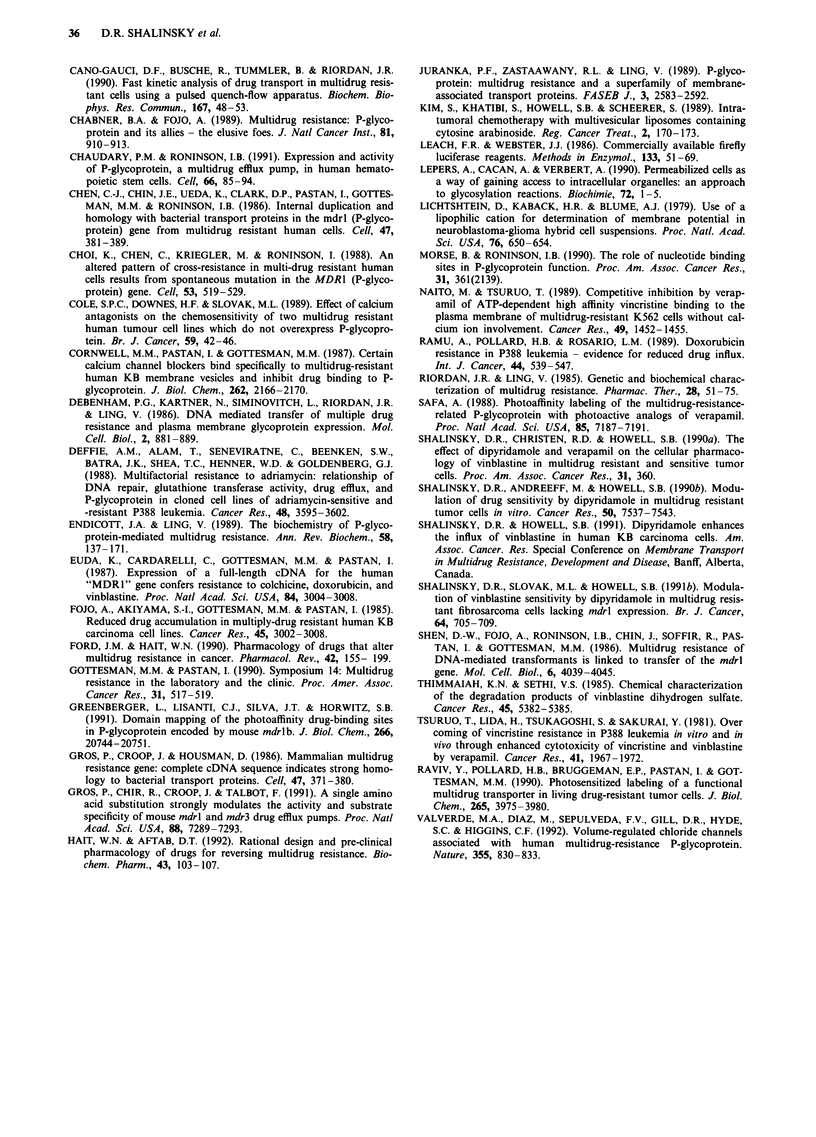# Regulation of initial vinblastine influx by P-glycoprotein.

**DOI:** 10.1038/bjc.1993.6

**Published:** 1993-01

**Authors:** D. R. Shalinsky, A. P. Jekunen, J. E. Alcaraz, R. D. Christen, S. Kim, S. Khatibi, S. B. Howell

**Affiliations:** Department of Medicine, University of California, San Diego, La Jolla 92093-0812.

## Abstract

P-glycoprotein (PGP) is an energy-dependent efflux pump that serves to protect cells against the cytotoxicity of many natural product drugs including vinblastine (VBL). In this study we investigated the role of PGP in regulating initial VBL influx. The apparent influx of VBL, measured over the first 20 s, was 2-fold lower in KB-GRC1 cells expressing a transfected mdr1 gene at high level than in non-expressing parental KB-3-1 cells. Inhibition of PGP efflux function with dipyridamole increased the influx rate constant by 4.0-fold in the KB-GRC1 cells but only 2.1-fold in the KB-3-1 cells. Verapamil, another inhibitor of PGP-mediated efflux, increased the initial influx rate constant by 2.7-fold in the KB-GRC1 cells but only 1.4-fold in the KB-3-1 cells. Inhibition of PGP function by depletion of ATP increased influx by 6.8-fold and 2.2-fold in the two cell types, respectively. Mutation of PGP at both ATP binding sites abolished its ability to limit initial influx. Thus, VBL is serving as an efficient substrate for the efflux pump even within the first few seconds of drug exposure, consistent with the hypothesis that PGP may directly efflux drug from the cell membrane.


Br. J. Cancer (1993), 67, 30–36 © Macmillan Press Ltd., 1993

### Regulation of initial vinblastine influx by P-glycoprotein

D.R. Shalinsky[1], A.P. Jekunen[1], J.E. Alcaraz[2], R.D. Christen[1], S. Kim[1], S. Khatibi[1] & S.B. Howell[1]

[1]*Laboratory of Pharmacology, Department of Medicine and the Cancer Center, and* [2]*Department of Biostatistics, University of California, San Diego, 9500 Gilman Drive, La Jolla, California 92093-0812, USA.*

**Summary** P-glycoprotein (PGP) is an energy-dependent efflux pump that serves to protect cells against the cytotoxicity of many natural product drugs including vinblastine (VBL). In this study we investigated the role of PGP in regulating initial VBL influx. The apparent influx of VBL, measured over the first 20 s, was 2-fold lower in KB-GRC1 cells expressing a transfected *mdr*1 gene at high level than in non-expressing parental KB-3-1 cells. Inhibition of PGP efflux function with dipyridamole increased the influx rate constant by 4.0-fold in the KB-GRC1 cells but only 2.1-fold in the KB-3-1 cells. Verapamil, another inhibitor of PGP-mediated efflux, increased the initial influx rate constant by 2.7-fold in the KB-GRC1 cells but only 1.4-fold in the KB-3-1 cells. Inhibition of PGP function by depletion of ATP increased influx by 6.8-fold and 2.2-fold in the two cell types, respectively. Mutation of PGP at both ATP binding sites abolished its ability to limit initial influx. Thus, VBL is serving as an efficient substrate for the efflux pump even within the first few seconds of drug exposure, consistent with the hypothesis that PGP may directly efflux drug from the cell membrane.

Overexpression of the *mdr*1 gene coding for the production of a 170 kd glycoprotein, PGP[3], is often a prominent feature of the MDR phenotype (Endicott & Ling, 1989; Juranka *et al.*, 1989). Transfection of the *mdr*1 gene into drug-sensitive clones confers drug resistance to cells that overexpress PGP (Debenham *et al.*, 1986; Shen *et al.*, 1986; Euda *et al.*, 1987). The transmembrane location, energy dependence, correlation with resistance and extensive homology of the *mdr*1 product with bacterial transport proteins is consistent with PGP acting as an energy-dependent efflux pump (Juranka *et al.*, 1989; Beck, 1987; Gros *et al.*, 1986; Chen *et al.*, 1986). A major strategy in reversing the MDR phenotype is to utilise agents that inhibit PGP function (Ford & Hait, 1990; Tsuruo *et al.*, 1981). For example, VPL's ability to inhibit drug efflux in many tumour types has been attributed to its ability to bind to PGP (Cornwell *et al.*, 1987; Safa, 1988; Naito & Tsuruo, 1989), but the modulation is absent in MDR cell lines that do not overexpress the *mdr*1 gene as their primary mechanism of resistance (Cole *et al.*, 1989).

There are a few reports indicating that *mdr*1 overexpression may be associated with a change in drug influx (Ramu *et al.*, 1989), or may fail to alter efflux (Deffie *et al.*, 1988), and modulators of the MDR phenotype such as VPL do not inhibit drug efflux in every MDR cell line overexpressing *mdr*1 (Fojo *et al.*, 1985). These data are difficult to interpret in light of the commonly accepted role of PGP as an efflux transporter. PGP shares extensive sequence homology with two major classes of transporters: (1) the multicomponent periplasmic protein-binding permeases that import substrates and (2) the hemolysin B-like proteins that export substrates (Juranka & Ling, 1989; Gross *et al.*, 1986; Chen *et al.*, 1987; Ames Ferro-Luzzi, 1986). The homology with proteins that transport substrates in and out of cells suggests that PGP is related to both types of transporters, and raises the question of whether PGP might have an influx function in addition to its efflux role (Riordan & Ling, 1985).

We have observed that overexpression of PGP decreases the initial influx of VBL over the first 20 s employing drug-sensitive KB-3-1 and MDR KB-GRC1 cells*. KB-GRC1 cells theoretically differ from KB-3-1 cells only by expression of PGP following transfection of *mdr*1 (Choi *et al.*, 1988), and are 67, 9.4, 4.5 and 1.5-fold cross-resistant to VBL, doxorubicin, colchicine and etoposide, respectively (*ibid.*, Shalinsky *et al.*, 1990*a*). Inhibition of PGP efflux function with two pharmacologic agents, DPM and VPL, increased initial influx. Reduction in efflux pump activity by depletion of ATP or mutation at the ATP binding sites also increased initial VBL influx. We conclude that PGP functions to decrease the apparent initial influx of VBL, and this decrease is mediated by the ability of PGP to efflux drug. Thus, VBL is serving as an efficient substrate for the efflux pump even within the first few seconds of drug exposure, consistent with the hypothesis that PGP directly effluxes drug from the cell membrane.

### Materials and methods

### *Drugs and chemicals*

DPM was a gift from Boehringer Ingelheim Pharmaceuticals, Inc. (Ridgefield, CT). DPM was dissolved in absolute ethanol to produce a stock concentration of 9.9 mM. G-418 sulfate was purchased from GIBCO (Grand Island, NY). VBL sulfate was obtained from Eli Lilly & Co. (Indianapolis, IN). VPL hydrochloride was purchased from American Regent Laboratories, Inc. (Shirley, NY). $NaN_3$ and the Bioluminescent Somatic Cell Assay Kit (FL-ASC) were purchased from Sigma Chemical Co. (St. Louis, MO). Stock solutions of these drugs were made by dissolving them in saline. Working solutions were prepared by further dilution in tissue culture medium. Propidium iodide was obtained from Calbiochem Co. (La Jolla, CA). $TPP^+$ (97% pure) was purchased from Aldrich Chemical Co. (Milwaukee, WI). [$^3$H]-$TPP^+$ (23 Ci $mmol^{-1}$) was purchased from Amersham Radiopharmaceuticals Inc. (Arlington Heights, IL) and stored at −20°C in ethanol.

[$^3$H]-VBL (10–20 Ci $mmol^{-1}$) in methanol was purchased under a special quality-control contract to ensure high purity from Moravek Biochemicals (Brea, CA), stored in the dark at −80°C and protected from light during experiments. The purity of [$^3$H]-VBL as a single peak was confirmed by HPLC

Correspondence: D.R. Shalinsky, Ligand Pharmaceuticals, 11149 N. Torrey Pines Rd., La Jolla, CA 92037, USA.
The abbreviations used are: $C_{ss}$, steady-state concentration; DPM, dipyridamole; G418-sulfate, Geneticin$^R$; $IC_{50}$, concentration of drug which inhibits colony formation by 50%; MDR, multidrug resistant or resistance; PGP, P-glycoprotein; PBS, phosphate buffered saline; $NaN_3$, sodium azide; $TPP^+$, tetraphenylphosphonium$^+$ bromide; VPL, verapamil hydrochloride; VBL, vinblastine sulfate.
Received 7 April 1992; and in revised form 27 July 1992.

*Portions of this work have been published in abstract form (Shalinsky *et al.*, 1990*a*; Shalinsky & Howell, 1991).

analysis according to the method of Thimmaiah and Sethi (1985). [$^3$H]-VBL was stable for at least 2 months when stored at −80°C. In addition, HPLC analysis of [$^3$H]-VBL extracted from cellular homogenates indicated no decomposition under the experimental conditions employed in these studies. The final specific activity of the [$^3$H]-VBL used for drug accumulation studies was 6.67 mCi $\mu$mol$^{-1}$.

*Cell lines and culture medium*

The drug sensitive KB-3-1 line (Akiyama *et al.*, 1985) and its multidrug resistant subline, KB-GRC1, were obtained from Dr. Igor Roninson (University of Illinois, College of Medicine, Chicago, IL). The KB-GRC1 line was derived by transfection of the wild-type *mdr*1 gene coupled to a Moloney Murine Leukemia Virus long terminal repeat into KB-3-1 cells (Choi *et al.*, 1988). Routine culture of these cells has been previously described (Shalinsky *et al.*, 1990*b*).

Mouse L cell variants, KK, MM and NEO (Morse & Roninson, 1990), were also obtained from Dr Roninson. KK cells were produced by transfection of the human wild-type *mdr*1 gene into parental fibroblast mouse L cells. MM cells were produced by transfection of a non-functional form of the *mdr*1 gene containing mutations at the ATP binding sites into parental fibroblast mouse L cells; specifically, lysine was mutated to methionine at positions 433 and 1076 in the amino acid sequence. NEO cells were produced by transfection of the vector without the *mdr*1 insert into parental fibroblast cells and represented the transfectant control cell line. Overexpression of functional and non-functional PGP in KK and MM cells was confirmed by staining of the respective cell ine with the monoclonal P-glycoCHEK C219 antibody (Centocor, Inc., Malvern, PA), and by measuring drug uptake and sensitivity. L cell variants were grown in the presence of 0.4 mg ml$^{-1}$ G-418 sulfate. All cells were grown at 37°C under 5% $CO_2$ in air in T25 or T75 tissue culture flasks (Corning Glass Works, NY).

*Modulation of cellular pharmacology*

Six nM [$^3$H]-VBL and modulator (20 $\mu$M) were rapidly added to subconfluent monolayer cultures in 60-mm dishes in 2 ml of culture medium equilibrated overnight in the $CO_2$ incubator at 37°C as described (*ibid.*). At appropriate time points, the medium was aspirated and the cells were washed (3 × 5 ml) with ice cold PBS (Oxoid, Columbia, MD). Incubations were carried out in the $CO_2$ incubator at 37°C for all time points $\geqslant$1 min. For time points $\leqslant$1 min, incubations were carried out using medium pre-equilibrated in the $CO_2$ incubator at 37°C. Time points were recorded from the addition of [$^3$H]-VBL alone or with modulator to the first aspiration. The average time recorded from addition of 2 ml of radiolabelled medium to the dish until aspiration was 0.89 ± 0.25 s, and uptake experiments under the first min were highly reproducible with linear rate constants being obtained ($r^2 \geqslant 0.93$). The cells were then digested overnight with 1 N NaOH, and neutralised aliquots were used for determination of protein content and cell associated radioactivity.

The percent of intracellular [$^3$H]-VBL that was in a bound form during uptake was determined by exposing monolayer cells to [$^3$H]-VBL for 1 h, washing the cells in PBS, resuspending in 1 ml of PBS and scraping with rubber policeman for transfer into 15 ml tubes. The cellular suspension was sonicated three times for 10 s on ice to destroy cellular membranes using a Vibra-cell (VC-50) sonicator (Sonics & Materials, Inc., Danbury, CT) set to 25 watts-/60 amplitude-vibrations. An aliquot was removed, digested in 1 N NaOH, neutralised with equimolar HCl, and counted by liquid scintillation spectroscopy to determine total radioactivity. The rest of the sonicated suspension was filtered through Centrifee$^R$ micropartition filters (Amicon, Beverly, MA) and the ultrafilterable [$^3$H]-VBL was counted by liquid scintillation spectroscopy. The percent of bound drug was calculated by subtracting the free ultrafiltrable amount of [$^3$H]-VBL from the total.

Efflux was determined after incubation of cells with 6 nM [$^3$H]-VBL for 2 h until steady-state had been reached. The medium was aspirated, cells were washed with ice cold PBS, and 2 ml of fresh VBL-free 37°C normal medium was added back to the dishes. DPM or VPL was supplemented to this medium to study the effect of these modulators on efflux. At designated time points, the fresh medium was aspirated and the cells washed with ice cold PBS. The actual time between addition of fresh medium and final aspiration was recorded in each case. Efflux was monitored from 30 s to 3 h.

*Measurement of cellular ATP levels*

Monolayers KB-GCR1 and KB-3-1 cells were pretreated for 2 h with varying concentrations of $NaN_3$ to deplete cellular ATP levels. After washing with ice-cold PBS, the cells were scraped from the dish and transferred to a plastic test tube as a suspension of $1-10 \times 10^6$ cells ml$^{-1}$. ATP levels were measured using fire fly luciferase with the Bioluminescent Somatic Cell Assay Kit [Sigma Chemical Co., St. Louis, MO; (Leach, 1986)]. Light emission was monitored using a Monolight bioluminescent luminometer (Model 2001, Analytical Luminescence Laboratory, San Diego, CA). A standard ATP curve was run with each experiment to ensure that the concentration of ATP and resulting emission of light was in the linear range. Data were expressed as light units relative to control for each experiment.

*Regression analysis of initial efflux and influx*

In this study, initial efflux was defined as efflux over the first 120 s of incubation in VBL-free medium. Initial influx was defined as the uptake over the first 20 s. The apparent unidirectional influx and initial efflux rate constants in KB-GRC1 and KB-3-1 cells were linear up to 240 s ($r^2 \geqslant 0.93$).

The rate constant for initial efflux was determined by fitting a line to the efflux data over the first 120 s. Several experiments, or runs, were performed under the same combination of cell line and condition (control, VPL or DPM); the equation for the i-th run was $C_i(t) = A_i[l\text{-}k_e t]$, where $C_i(t)$ is the concentration of VBL at any time t, $A_i$ is the initial concentration of VBL at $C_{ss}$ prior to efflux, as shown by the fitted y-intercept, and $k_e$ is the rate constant for initial efflux. Simultaneous fitting of the data from all runs was done to obtain a single estimate of $K_e$; its standard error and degrees of freedom appeared in the regression output. The statistic $r^2$ for these fittings ranged from 0.983 to 0.998 across the various combinations of cell line and condition. Similarly, for each combination of cell line and condition, the rate constant for influx was determined by fitting a line to the influx data over the first 20 s of influx. The equation for the i-th run was $C_i(t) = A_i[l + k_{in}t]$, where $C_i(t)$ is the time zero binding of VBL, as shown by the fitted y-intercept, and $k_{in}$ is the rate constant for initial uptake, i.e. influx. The statistic $r^2$ for these fittings ranged from 0.930 to 0.978 across the various combinations of cell line and condition. The fitted rate constants for efflux and influx were compared across the control, VPL and DPM groups using the Satterwaite modification of Student's *t* test.

*Statistical analysis*

Unless othersise noted, the data were expressed as the group mean ± SE of duplicate determinations from each of *n* experiments. The Student's *t* test for grouped data were used unless otherwise stated. In all cases, significance was at the level of $P < 0.05$.

### Results

*Effect of PGP on VBL influx*

The KB-GRC1 and KB-3-1 cell lines were employed because they permitted a direct comparison of the effect of PGP on

VBL uptake without the confounding effects of other resistance mechanisms associated with cells selected with increasing concentrations of drug (Chabner & Fojo, 1989). Figure 1a shows the time course of [$^3$H]-VBL uptake in the parental KB-3-1 and PGP-expressing KB-GRC1 cells. KB-GRC1 cells accumulated VBL at a lower rate and to a smaller extent than the parental KB-3-1 cells, consistent with the presence of the PGP efflux transporter in KB-GRC1 cells. Expression of PGP only in the KB-GRC1 cells was confirmed by staining of the cells with the C219 monoclonal antibody followed by flow cytometric analysis (data not shown). The $C_{ss}$ or VBL under control conditions was reached by 20 and 60 min in KB-GRC1 and KB-3-1 cells, respectively. The $C_{ss}$ was 5.2-fold lower in the KB-GRC1 than KB-3-1 cells. Figure 1b also shows the time course of efflux of [$^3$H]-VBL. The KB-GRC1 cells had a more rapid and extensive loss of [$^3$H]-VBL. The efflux data over the first 120 s were well fit by a first order decay curve from which the initial efflux rate constant was calculated ($r^2 \geqslant 0.983$). As shown in Table I, the rate constant for initial efflux was 2.5-fold higher in KB-GRC1 than KB-3-1 cells, confirming the presence of the PGP efflux transporter in the KB-GRC1 cells. After 10 min, KB-GRC1 cells had effluxed 53% of the drug they contained at steady-state but KB-3-1 cells had effluxed only 24% of their steady-state content. After 3 h, only 10% of the steady-state level of VBL remained in KB-GRC1 cells compared to 50% in the KB-3-1 cells. These results establish that the KB-GRC1 cells have the transport characteristics expected as a result of high level expression of a PGP efflux pump.

However, in addition to changes in $C_{ss}$ and efflux, there was a large difference in the initial influx of [$^3$H]-VBL between the KB-GRC1 and KB-3-1 cells measured over the first few seconds of drug exposure (Figure 2). The initial influx data were well fit by a straight line ($r^2 \geqslant 0.930$) from which the influx rate constant was calculated. As shown in Table II, the rate constant for influx was 4.2-fold lower in KB-GRC1 relative to KB-3-1 cells. A statistically significant difference in uptake existed even at 5 s after the start of the exposure ($P < 0.05$). Figure 2 also shows that the influx was completely abolished when the cells were incubated at 4°C on ice, indicating that diffusion of VBL into cells was stopped at low temperature. Initial influx was not saturable in either cell line at concentrations up to 324 μM (data not shown). These data that show that expression of PGP results in a lower initial influx rate, suggesting that PGP was able to rapidly efflux VBL during the initial moments of exposure.

**Figure 1** Uptake **a**, and efflux **b**, of [$^3$H]-VBL in KB-GRC1 (O——O) and KB-3-1 (●——●) cells under control conditions. Cells were loaded with [$^3$H]-VBL for 2 h prior to efflux. Each point is plotted as the mean ± s.e. of 3–4 experiments.

**Table I** Rate constants for initial efflux of VBL over the first 120 s of efflux

| Cell line | Condition | *Rate ± s.e.*[a] *(min$^{-1}$)* | *P value compared to Control* | *P value compared to VPL* |
|---|---|---|---|---|
| KB-GRC1 | Control[b] | 0.130 ± 0.011 | | |
| | VPL (20 μM) | 0.112 ± 0.012 | 0.308 | |
| | DPM (20 μM) | 0.074 ± 0.013 | 0.009 | 0.052 |
| KB-3-1 | Control | 0.052 ± 0.014 | | |
| | VPL (20 μM) | 0.047 ± 0.004 | 0.765 | |
| | DPM (20 μM) | 0.030 ± 0.005 | 0.193 | 0.026 |

[a]Data are expressed as fitted estimate of the rate constant. Values are mean ± s.e from three experiments. [b]KB-GRC1 *vs* KB-3-1 control: *P*-value = 0.002.

**Figure 2** Influx of [$^3$H]-VBL in KB-GRC1 (O——O) and KB-3-1 (●——●) cells under control conditions at 37°C. Influx was also monitored at 4°C in the same cells (KB-GRC1, O···O; KB-3-1, ●···●). Each point is plotted as the mean ± s.e. of 3–7 experiments.

**Table II** Rate constants for influx of VBL over the first 20 s of drug exposure

| Cell line | Condition | *Rate ± s.e.*[a] *(× $10^{-5}$ s$^{-1}$)* | *P value compared to Control* | *P value compared to VPL* |
|---|---|---|---|---|
| KB-GRC1 | Control[b] | 0.393 ± 0.017 | | |
| | VPL (20 μM) | 1.042 ± 0.137 | 0.001 | |
| | DPM (20 μM) | 1.576 ± 0.297 | 0.002 | 0.119 |
| KB-3-1 | Control | 1.634 ± 0.053 | | |
| | VPL (20 μM) | 2.245 ± 0.228 | 0.069 | |
| | DPM (20 μM) | 3.413 ± 0.372 | 0.013 | 0.013 |

[a]Data are expressed as fitted estimate of the rate constant. Value are mean ± s.e. from 5–7 experiments. [b]KB-GRC1 *vs* KB-3-1 control: *P*-value < 0.001.

*Effect of DPM on VBL influx*

If the effect of PGP on initial influx was dependent on its efflux pump activity, then inhibition of the pump with pharmacologic agents such as DPM should increase initial influx. As shown in Figure 3, DPM produced an instantaneous doubling of the time zero binding of [$^3$H]-VBL in each cell line. Pretreatment for up to 1 h did not alter the magnitude

**Figure 3** Influx of [$^3$H]-VBL in KB-GRC1 **a**, and KB-3-1 **b**, cells in the presence of 20 μM DPM or VPL. Influx is shown under control conditions (○——○), with DPM (■——■) and VPL (□——□). Each point is plotted as the mean ± s.e. of 5–7 experiments.

**Figure 4** Efflux of [$^3$H]-VBL in KB-GRC1 **a**, and KB-3-1 **b**, cells. Cells were preloaded with radiolabelled VBL for 2 h prior to efflux under control conditions (○——○), or in the presence of 20 μM DPM (●——●) or VPL (▲——▲). Points are plotted as the mean ± s.e. of three experiments.

of this effect (data not shown). The ability of DPM to increase initial influx was determined by regression analysis of the data over the first 20 s of incubation. These data were well fit to a straight line ($r^2 \geqslant 0.930$). Table II shows that DPM increased the rate constant for influx by 4.0 and 2.1-fold in KB-GRC1 and KB-3-1 cells, respectively ($P \leqslant 0.013$). DPM enhanced the influx in a dose-dependent saturable manner to an equivalent level in both KB-GRC1 and KB-3-1 cell lines (data not shown). The concentration of DPM required to produce a half-maximal increase in influx was 3 μM in KB-GRC1 cells and it was 1 μM in KB-3-1 cells. Saturation of influx occurred at concentrations ⩾ 20 μM. Concentrations of DPM ⩽ 200 μM did not alter cellular ability to exclude trypan blue under these conditions. Measurement of the extent of bound intracellular VBL demonstrated that 55% of the radiolabel was ultrafiltrable after a 1 h incubation. Twenty μM DPM did not change the level of ultrafilterable [$^3$H]-VBL in two experiments, indicating that the DPM-induced increase in 1 h accumulation was not due to a change in a tightly-bound fraction of VBL to proteins of ⩽ 25,000 molecular weight. Thus, this inhibitor of PGP did in fact increase initial influx.

That DPM inhibited efflux pump activity was shown by its effect on the time course of VBL efflux. Figure 4 shows that DPM inhibited the efflux [$^3$H]-VBL in both the KB-GRC1 and KB-3-1 cells with the most prominent effects observed in KB-GRC1 cells. DPM produced a statistically significant decrease in both the initial efflux rate constant (Table I) and the terminal rate constant from 0.00606 ± 0.00057 min$^{-1}$ (SE) to 0.00005 ± 0.00037 (SE) min$^{-1}$ ($P < 0.00001$) in KB-GRC1 cells. This is reflected by the fact that DPM almost completely blocked the efflux of [$^3$H]-VBL after 30 min. Further experiments indicated that the [$^3$H]-VBL remaining in the cell after 1 h of efflux in DPM-containing medium was readily effluxable if DPM was removed from the efflux medium (data not shown), confirming that DPM did not lock VBL into an irreversibly-bound intracellular compartment. In KB-3-1 cells, DPM did not significantly reduce the initial efflux or terminal rate constants ($P \geqslant 0.193$), but still had a significant effect on decreasing efflux by 1.9-fold ($P = 0.026$) as shown in Figure 4.

Consistent with its ability to both increase influx and inhibit efflux, DPM produced a large increase in the $C_{ss}$ in KB-GRC1 cells that plateaued at a level 9.2-fold above control at steady-state (data not shown). In comparison, DPM elevated the $C_{ss}$ by only 1.8-fold in KB-3-1 cells, but the enhancement was statistically significant ($P < 0.05$, paired *t* test).

Further experiments were conducted to examine whether the effects of DPM were due to permeabilisation of the cellular membrane. DPM did not affect the uptake or efflux of [$^3$H]-VBL in protein-free liposomes (data not shown) prepared as previously described (Kim *et al.*, 1989). In flow cytometric studies, DPM did not alter the forward light scatter of KB-3-1 cells, but the membrane permeabilising agent, digitonin (Lepers *et al.*, 1990), produced a maximal increase within 75 s of incubation (data not shown). Digitonin, but not DPM, also produced a large increase in permeability to propidium iodide as determined by measuring cellular fluorescence of this agent (data not shown). Furthermore, DPM did not alter the membrane potential in KB-3-1 cells as determined by measuring the accumulation of [$^3$H]-TPP$^+$ at 2 h (Lichtshtein *et al.*, 1979). These experiments provided evidence that DPM did not permeabilise cellular membranes.

As shown in Figure 2, there was a high time zero binding of [$^3$H]-VBL in tumour cells that was modulatable by DPM. Liposomes were used as a model to determine whether this initial binding was unique to cell membranes. Liposomes incubated at 37°C had an initial time zero association of 3404 ± 257 (s.d.) c.p.m.; this value was reduced to 2267 ± 505 (s.d.) c.p.m. for liposomes incubated on ice at 4°C, respectively ($n = 2$). The modest decrease observed in time zero binding with reduction in temperature in both liposomes and cells, and the fact that significant time zero binding occurred in both liposomes and cells suggested that the time zero binding represents non-specific association of VBL with membrane lipids.

### *Effect of VPL on VBL influx*

Table II shows that VPL produced a 2.7-fold increase in the rate constant for initial influx in KB-GRC1 cells but only a statistically insignificant 1.4-fold increase in KB-3-1 cells. The influx data over the first 20 s of incubation were well fit to a straight line ($r^2 \geqslant 0.930$). VPL produced a 4.6-fold increase in the $C_{ss}$ of [$^3$H]-VBL in KB-GRC1 cells but the 1.3-fold increase in $C_{ss}$ in KB-3-1 cells was statistically insignificant (data not shown). As shown in Table I, VPL did not significantly inhibit initial efflux in either cell line, in agreement with a previously published report (Fojo *et al.*, 1985). Thus, VPL had more pronounced effects on initial influx than on initial efflux, but the greatest effect was in *mdr*1-overexpressing KB-GRC1 cells. Thus, this inhibitor of PGP activity preferentially increased influx as compared to inhibiting efflux.

### *Effect of mutation of PGP on VBL influx*

Further experiments were performed to determine the effect on VBL influx of inactivating PGP by mutating ATP binding sites. L cells were transfected with wild-type or mutationally inactivated *mdr*1, and an empty vector to produce the KK, MM and NEO sublines, respectively. The KK, MM and NEO cell ines had a $C_{ss}$ of 0.57 ± 0.26 (s.d.), 2.49 ± 0.10 (s.d.) and 2.07 ± 0.49 (s.d.) pmol VBL mg$^{-1}$ protein, respectively ($n$ = 2–3), and respective $IC_{50}$ values in the clonogenic assay of 166 ± 23 (s.d.), 25 ± 5 and 22 ± 3 (s.d.) nM for VBL ($n$ = 3–5, data not shown). KK cells were also 5-fold resistant to colchicine compared to MM and NEO cells[†], consistent with the presence of abundant functional PGP uniquely in KK cells. Staining with the monoclonal antibody C219 showed a high level of staining for PGP in the KK and MM cell lines and no detectable level in NEO cells (data not shown). While NEO cells express a low level of endogenous PGP[†], they served as an appropriate control for the KK and MM cells, in which the transfection conferred detectable levels of PGP and in the case of KK, an observable MDR phenotype. In this model, the initial influx of VBL was reduced in the KK cells documented to possess the functional form of PGP relative to the other cell lines (Figure 5). The presence of a high level of non-functional PGP did not alter the influx rate in MM cells relative to the transfectant control NEO cell line.

### *Effect of energy depletion on VBL influx*

Since PGP is an energy-dependent pump, ATP depletion would be expected to increase influx selectively in KB-GRC1 cells if PGP was responsible for the differences in influx observed between KB-GRC1 and KB-3-1 cells. Therefore, cellular ATP content was depleted. Table III shows that $NaN_3$ depleted cellular ATP levels in a dose-dependent manner. Cellular viability was ⩾94% after exposure to $NaN_3$, and this treatment did not affect efficiency when the cells were subsequently seeded in normal medium (data not shown). Figure 6 shows that pretreatment for 2 h with 100 mM $NaN_3$ increased the influx of VBL by 6.8 and 2.2-fold in KB-GRC1 and KB-3-1 cells, respectively. The fact that ATP depletion equalised the influx rate indicated that the efflux pump activity of PGP was responsible for the lower rate even at times ⩽20 s. These data supported the hypothesis that the efflux function of PGP was fully active even at the earliest time intervals and indicated that effects on the apparent influx truly represented inhibition of rapid efflux, and that one could not interpret the influx period to truly represent zero-trans influx in the KB-GRC1 cells.

**Figure 5** Influx of [$^3$H]-VBL in mouse L cell variants, KK (●——●), MM (■——■), and NEO (O——O). Points mean ± s.e. of three experiments.

**Table III** Depletion of ATP levels by $NaN_3$ in KB-GRC1 and KB-3-1 cells

| Cell line | *Per cent ATP remaining after 2 h exposure to $NaN_3$* *$NaN_3$ concentration (mM)* 10 | 50 | 100 |
|---|---|---|---|
| KB-GRC1 | 49.4 ± 25.0[a] | 21.1 ± 1.4 | 19.6 ± 4.8 |
| KB-3-1 | 59.9 ± 16.1 | 20.6 ± 2.1 | 13.1 ± 7.0 |

[a]Value are expressed as mean ± s.d. of two experiments performed in duplicate as described in Materials and methods.

**Figure 6** Initial influx of [$^3$H]-VBL in KB-GRC1 **a**, and KB-3-1 **b**, cells. Influx was monitored under normal energy-replete conditions (O——O) and under energy-depleted conditions produced by pre-incubation with 100 mM $NaN_3$ (●——●). Points are mean ± s.e. of three experiments.

[†]I.B. Roninson, personal communication.

### Discussion

Cells that overexpress the *mdr*1 gene have decreased steady-state accumulation and increased efflux of many anticancer drugs including VBL (Endicott & Ling, 1989; Juranka *et al.*, 1989). The product of the *mdr*1 gene, PGP, is thought to function as an energy-dependent efflux pump, and drugs such as VPL are believed to reverse the MDR phenotype by virtue of their ability to bind to PGP and inhibit efflux activity (reviewed in Ford & Hait, 1990). KB-GRC1 and KB-3-1 cells theoretically differ from each other only by virtue of the presence of overexpressed amounts of PGP in the former cells (Choi *et al.*, 1988), and as anticipated, the KB-GRC1 cells accumulated less VBL at steady-state, and effluxed VBL more rapidly. KB-GRC1 cells, however, also demonstrated a much lower rate of initial VBL influx than KB-3-1 cells.

During the first few seconds of VBL influx, one would expect that the cytosolic concentration of VBL would be at its lowest level relative to the levels that would be achieved over time, and thus that efflux would be relatively unimportant and incapable of influencing the rate of initial influx. However, the presence of PGP in the membrane did decrease initial influx measured over the first few seconds. Three possible explanations present themselves. The first and second possibilities are that the presence of PGP in the membrane decreases diffusional permeability to VBL, or decreases its intracellular trapping. Favouring this is the fact that PGP is known to export acids (Gros *et al.*, 1986; Chen *et al.*, 1987; Ames Ferro-Luzzi, 1986) and may serve as a chloride channel (Valverde *et al.*, 1992), and thus could indirectly alter the intracellular environment to decrease drug trapping. The third possibility is that PGP may be effluxing VBL directly from the cell membrane or cytoplasm under conditions which are not zero-trans.

Several lines of evidence favour the latter argument. First, when the efflux function of PGP was inhibited by DPM, an MDR modulator that binds to PGP to produce synergy with VBL (Asoh *et al.*, 1989; Shalinsky *et al.*, 1990*b*; Shalinsky *et al.*, 1991*b*), initial influx increased, and did so to a greater degree in the KB-GRC1 cells over-expressing PGP than in the KB-3-1 cells that do not express detectable amounts by Northern or polymerase chain reaction analyses of *mdr*1 mRNA (Akiyama *et al.*, 1985; Chaudary & Roninson, 1991). That DPM can function as an inhibitor of the efflux activity of the PGP was shown by the fact that it produced a statistically significant reduction in the initial efflux of VBL from preloaded cells. The fact that DPM inhibited efflux and did not alter membrane permeability indicates that its actions were specifically and primarily targeted to inhibiting PGP efflux activity. Like DPM, VPL also increased initial influx, and did so to a greater extent in KB-GRC1 than KB-3-1 cells. Although the effect of VPL on inhibiting initial efflux did not reach the level of statistical significance, VPL has been shown to be an effective inhibitor of the efflux function of PGP in numerous other studies (Ford & Hait, 1990). Second, when the efflux pump function of PGP was eliminated by ATP depletion, the initial influx rate increased, and the effect was larger in the cells expressing the greater amount of PGP. Third, while mouse L cells transfected with a functional form of the *mdr*1 gene demonstrate a decrease in initial influx compared to control cells transfected with the same vector not containing the *mdr*1 gene, cells transfected with a mutated *mdr*1 producing a nonfunctional PGP do not have impaired initial influx. Thus, the presence of large amounts of PGP containing only a single amino acid substitution in each of the ATP binding sites was not by itself able to alter initial influx, arguing that simply the presence of the protein in the membrane did not change diffusional permeability to VBL.

While these kinetic studies could not distinguish between efflux from the membranal or cytoplasmic compartments, we believe that the data are most consistent with the hypothesis that PGP can export substrates directly from the cellular membrane. In support of this concept is the rapidity with which efflux activity was observed and inhibited, and the fact that most substrates and modulators of PGP activity are lipophilic (Ford & Hait, 1990) and thus would be expected to interact more highly within the lipid membrane bilayer. Furthermore, reports in the literature indicate that PGP serves as a hydrophobic membrane 'vacuum cleaner' to export drug from the cellular membrane (Gros *et al.*, 1986; Raviv *et al.*, 1990; Gottesman & Pastan, 1990). A more prominent role for the membranal rather than cytoplasmically-bound substrate is invoked by the work of Raviv *et al.* (1990) who have reported that doxorubicin interacts with a site on PGP within the cellular membrane. In addition, identification of putative binding sites close to or within the transmembrane regions of PGP would also support this hypothesis (Choi *et al.*, 1988; Greenberger *et al.*, 1991; Gros *et al.*, 1991; Hait & Aftab, 1992).

If PGP can utilise VBL in the membrane compartment as a substrate, then what appears to be a reduction in initial influx is in fact due to the ability of the pump to rapidly efflux VBL. Other investigators have also demonstrated the ability of PGP to efflux VBL and doxorubicin within millisec to sec of exposure in MDR cells (Cano-Gauci *et al.*, 1990), and modulators of the MDR phenotype have been shown to inhibit PGP-chloride-associated channel activity within 30 s of exposure (Valverde *et al.*, 1992) providing further support for the concept than that PGP can efflux drug even after very short periods of drug exposure, and that modulators can act rapidly enough to account for their ability to increase initial uptake within a few seconds of addition to the culture.

In summary, this report demonstrates that the KB-GRC1 cell line presents a good model for the study of PGP function at short time intervals. Specifically, PGP-mediated efflux can be quantitated at short time points as can the efflux-modulating activity of MDR chemosensitisers such as DPM and VPL. These data are consistent with the hypothesis that PGP directly effluxes drug from the cellular membrane.

We thank Dr Igor Roninson and Mr Brian Morse for generously supplying the KB and L cell tumour lines, Boehringer Ingelheim, Inc. for the gift of DPM, and Centocor, Inc. for the gift of P-glycoCHEK. We also thank Mr Dennis Heath for expert HPLC analysis of VBL, and Mr Dennis Young for performing flow cytometric studies, and Drs I. David Goldman and William T. Beck for helpful discussions.

This work was supported by grant CA 23100 from the NIH, grant CH368 from the American Cancer Society, and grants from Boehringer Ingelheim Inc., Bristol-Myers Squibb Co, and a grant from the Cancer Research Coordinating Committee (CRCC) of the University of California. This work was conducted in part by the Clayton Foundation for Research – California Division. Drs Howell and Jekunen are Clayton Foundation Investigators. Dr Shalinsky was supported in part by NIH CA 09290, the CRCC and National Research Service Award CA 08993.

### References

AKIYAMA, S.-I., FOJO, A., HANOVER, J.A., PASTAN, I. & GOTTESMAN, M.M. (1985). Isolation and genetic characterization of human KB cell lines resistant to multiple drugs. *Somat. Cell Mol. Genetics*, **11**, 117–126.

AMES FERRO-LUZZI, G. (1986). Bacterial periplasmic transport systems: structure, mechanism, and evolution. *Ann. Rev. Biochem.*, **55**, 397–425.

ASHOH, K.-I., SABURI, Y., SATO, S.-I., NOGAE, I., KOHNO, K. & KUWANO, M. (1989). Potentiation of some anticancer agents by dipyridamole against drug-sensitive and drug-resistant cell lines. *Jpn. J. Cancer Res.*, **80**, 475–481.

BECK, W.T. (1987). The cell biology of multiple drug resistance. *Biochem. Pharm.*, **36**, 2879–2887.

CANO-GAUCI, D.F., BUSCHE, R., TUMMLER, B. & RIORDAN, J.R. (1990). Fast kinetic analysis of drug transport in multidrug resistant cells using a pulsed quench-flow apparatus. *Biochem. Biophys. Res. Commun.*, **167**, 48–53.

CHABNER, B.A. & FOJO, A. (1989). Multidrug resistance: P-glycoprotein and its allies – the elusive foes. *J. Natl Cancer Inst.*, **81**, 910–913.

CHAUDARY, P.M. & RONINSON, I.B. (1991). Expression and activity of P-glycoprotein, a multidrug efflux pump, in human hematopoietic stem cells. *Cell*, **66**, 85–94.

CHEN, C.-J., CHIN, J.E., UEDA, K., CLARK, D.P., PASTAN, I., GOTTESMAN, M.M. & RONINSON, I.B. (1986). Internal duplication and homology with bacterial transport proteins in the mdr1 (P-glycoprotein) gene from multidrug resistant human cells. *Cell*, **47**, 381–389.

CHOI, K., CHEN, C., KRIEGLER, M. & RONINSON, I. (1988). An altered pattern of cross-resistance in multi-drug resistant human cells results from spontaneous mutation in the *MDR*1 (P-glycoprotein) gene. *Cell*, **53**, 519–529.

COLE, S.P.C., DOWNES, H.F. & SLOVAK, M.L. (1989). Effect of calcium antagonists on the chemosensitivity of two multidrug resistant human tumour cell lines which do not overexpress P-glycoprotein. *Br. J. Cancer*, **59**, 42–46.

CORNWELL, M.M., PASTAN, I. & GOTTESMAN, M.M. (1987). Certain calcium channel blockers bind specifically to multidrug-resistant human KB membrane vesicles and inhibit drug binding to P-glycoprotein. *J. Biol. Chem.*, **262**, 2166–2170.

DEBENHAM, P.G., KARTNER, N., SIMINOVITCH, L., RIORDAN, J.R. & LING, V. (1986). DNA mediated transfer of multiple drug resistance and plasma membrane glycoprotein expression. *Mol. Cell. Biol.*, **2**, 881–889.

DEFFIE, A.M., ALAM, T., SENEVIRATNE, C., BEENKEN, S.W., BATRA, J.K., SHEA, T.C., HENNER, W.D. & GOLDENBERG, G.J. (1988). Multifactorial resistance to adriamycin: relationship of DNA repair, glutathione transferase activity, drug efflux, and P-glycoprotein in cloned cell lines of adriamycin-sensitive and -resistant P388 leukemia. *Cancer Res.*, **48**, 3595–3602.

ENDICOTT, J.A. & LING, V. (1989). The biochemistry of P-glycoprotein-mediated multidrug resistance. *Ann. Rev. Biochem.*, **58**, 137–171.

EUDA, K., CARDARELLI, C., GOTTESMAN, M.M. & PASTAN, I. (1987). Expression of a full-length cDNA for the human "MDR1" gene confers resistance to colchicine, doxorubicin, and vinblastine. *Proc. Natl Acad. Sci. USA*, **84**, 3004–3008.

FOJO, A., AKIYAMA, S.-I., GOTTESMAN, M.M. & PASTAN, I. (1985). Reduced drug accumulation in multiply-drug resistant human KB carcinoma cell lines. *Cancer Res.*, **45**, 3002–3008.

FORD, J.M. & HAIT, W.N. (1990). Pharmacology of drugs that alter multidrug resistance in cancer. *Pharmacol. Rev.*, **42**, 155– 199.

GOTTESMAN, M.M. & PASTAN, I. (1990). Symposium 14: Multidrug resistance in the laboratory and the clinic. *Proc. Amer. Assoc. Cancer Res.*, **31**, 517–519.

GREENBERGER, L., LISANTI, C.J., SILVA, J.T. & HORWITZ, S.B. (1991). Domain mapping of the photoaffinity drug-binding sites in P-glycoprotein encoded by mouse *mdr*1b. *J. Biol. Chem.*, **266**, 20744–20751.

GROS, P., CROOP, J. & HOUSMAN, D. (1986). Mammalian multidrug resistance gene: complete cDNA sequence indicates strong homology to bacterial transport proteins. *Cell*, **47**, 371–380.

GROS, P., CHIR, R., CROOP, J. & TALBOT, F. (1991). A single amino acid substitution strongly modulates the activity and substrate specificity of mouse *mdr*1 and *mdr*3 drug efflux pumps. *Proc. Natl Acad. Sci. USA*, **88**, 7289–7293.

HAIT, W.N. & AFTAB, D.T. (1992). Rational design and pre-clinical pharmacology of drugs for reversing multidrug resistance. *Biochem. Pharm.*, **43**, 103–107.

JURANKA, P.F., ZASTAAWANY, R.L. & LING, V. (1989). P-glycoprotein: multidrug resistance and a superfamily of membrane-associated transport proteins. *FASEB J.*, **3**, 2583–2592.

KIM, S., KHATIBI, S., HOWELL, S.B. & SCHEERER, S. (1989). Intratumoral chemotherapy with multivesicular liposomes containing cytosine arabinoside. *Reg. Cancer Treat.*, **2**, 170–173.

LEACH, F.R. & WEBSTER, J.J. (1986). Commercially available firefly luciferase reagents. *Methods in Enzymol.*, **133**, 51–69.

LEPERS, A., CACAN, A. & VERBERT, A. (1990). Permeabilized cells as a way of gaining access to intracellular organelles: an approach to glycosylation reactions. *Biochimie*, **72**, 1–5.

LICHTSHTEIN, D., KABACK, H.R. & BLUME, A.J. (1979). Use of a lipophilic cation for determination of membrane potential in neuroblastoma-glioma hybrid cell suspensions. *Proc. Natl. Acad. Sci. USA*, **76**, 650–654.

MORSE, B. & RONINSON, I.B. (1990). The role of nucleotide binding sites in P-glycoprotein function. *Proc. Am. Assoc. Cancer Res.*, **31**, 361(2139).

NAITO, M. & TSURUO, T. (1989). Competitive inhibition by verapamil of ATP-dependent high affinity vincristine binding to the plasma membrane of multidrug-resistant K562 cells without calcium ion involvement. *Cancer Res.*, **49**, 1452–1455.

RAMU, A., POLLARD, H.B. & ROSARIO, L.M. (1989). Doxorubicin resistance in P388 leukemia – evidence for reduced drug influx. *Int. J. Cancer*, **44**, 539–547.

RIORDAN, J.R. & LING, V. (1985). Genetic and biochemical characterization of multidrug resistance. *Pharmac. Ther.*, **28**, 51–75.

SAFA, A. (1988). Photoaffinity labeling of the multidrug-resistance-related P-glycoprotein with photoactive analogs of verapamil. *Proc. Natl Acad. Sci. USA*, **85**, 7187–7191.

SHALINSKY, D.R., CHRISTEN, R.D. & HOWELL, S.B. (1990*a*). The effect of dipyridamole and verapamil on the cellular pharmacology of vinblastine in multidrug resistant and sensitive tumor cells. *Proc. Am. Assoc. Cancer Res.*, **31**, 360.

SHALINSKY, D.R., ANDREEFF, M. & HOWELL, S.B. (1990*b*). Modulation of drug sensitivity by dipyridamole in multidrug resistant tumor cells *in vitro*. *Cancer Res.*, **50**, 7537–7543.

SHALINSKY, D.R. & HOWELL, S.B. (1991). Dipyridamole enhances the influx of vinblastine in human KB carcinoma cells. *Am. Assoc. Cancer. Res.* Special Conference on *Membrane Transport in Multidrug Resistance, Development and Disease*, Banff, Alberta, Canada.

SHALINSKY, D.R., SLOVAK, M.L. & HOWELL, S.B. (1991*b*). Modulation of vinblastine sensitivity by dipyridamole in multidrug resistant fibrosarcoma cells lacking *mdr*1 expression. *Br. J. Cancer*, **64**, 705–709.

SHEN, D.-W., FOJO, A., RONINSON, I.B., CHIN, J., SOFFIR, R., PASTAN, I. & GOTTESMAN, M.M. (1986). Multidrug resistance of DNA-mediated transformants is linked to transfer of the *mdr*1 gene. *Mol. Cell. Biol.*, **6**, 4039–4045.

THIMMAIAH, K.N. & SETHI, V.S. (1985). Chemical characterization of the degradation products of vinblastine dihydrogen sulfate. *Cancer Res.*, **45**, 5382–5385.

TSURUO, T., LIDA, H., TSUKAGOSHI, S. & SAKURAI, Y. (1981). Over coming of vincristine resistance in P388 leukemia *in vitro* and *in vivo* through enhanced cytotoxicity of vincristine and vinblastine by verapamil. *Cancer Res.*, **41**, 1967–1972.

RAVIV, Y., POLLARD, H.B., BRUGGEMAN, E.P., PASTAN, I. & GOTTESMAN, M.M. (1990). Photosensitized labeling of a functional multidrug transporter in living drug-resistant tumor cells. *J. Biol. Chem.*, **265**, 3975–3980.

VALVERDE, M.A., DIAZ, M., SEPULVEDA, F.V., GILL, D.R., HYDE, S.C. & HIGGINS, C.F. (1992). Volume-regulated chloride channels associated with human multidrug-resistance P-glycoprotein. *Nature*, **355**, 830–833.